# Higher LPA2 and LPA6 mRNA Levels in Hepatocellular Carcinoma Are Associated with Poorer Differentiation, Microvascular Invasion and Earlier Recurrence with Higher Serum Autotaxin Levels

**DOI:** 10.1371/journal.pone.0161825

**Published:** 2016-09-01

**Authors:** Kenichiro Enooku, Baasanjav Uranbileg, Hitoshi Ikeda, Makoto Kurano, Masaya Sato, Hiroki Kudo, Harufumi Maki, Kazuhiko Koike, Kiyoshi Hasegawa, Norihiro Kokudo, Yutaka Yatomi

**Affiliations:** 1 Department of Gastroenterology, The University of Tokyo, Tokyo, Japan; 2 Department of Clinical Laboratory Medicine, The University of Tokyo, Tokyo, Japan; 3 Hepato-Biliary-Pancreatic Surgery Division, Department of Surgery, The University of Tokyo, Tokyo, Japan; University of North Carolina at Chapel Hill School of Medicine, UNITED STATES

## Abstract

Hepatocellular carcinoma (HCC) commonly develops in patients with liver fibrosis; in these patients, the blood levels of lysophosphatidic acid (LPA) and its generating enzyme autotaxin (ATX) increase with the liver fibrosis stage. We aimed to examine the potential relevance of ATX and LPA in HCC. Fifty-eight HCC patients who underwent surgical treatment were consecutively enrolled in the study. Among the LPA receptors in HCC, higher LPA2 mRNA levels correlated with poorer differentiation, and higher LPA6 mRNA levels correlated with microvascular invasion, which suggested a higher malignant potential of HCC with increased LPA2 and LPA6 expression. In patients with primary HCC, neither LPA2 nor LPA6 mRNA levels were associated with recurrence. However, when serum ATX levels were combined for analysis as a surrogate for plasma LPA levels, the cumulative intra-hepatic recurrence rate was higher in patients in whom both serum ATX levels and LPA2 or LPA6 mRNA levels were higher than the median. However, the mRNA level of phosphatidic acid-selective phospholipase A1ɑ, another LPA-generating enzyme, in HCC patients was not associated with pathological findings or recurrence, even in combination with the expression of LPA receptors. Higher LPA2 mRNA levels were associated with poorer differentiation, and higher LPA6 levels were associated with microvascular invasion in HCC; both became a risk factor for recurrence after surgical treatment when combined with increased serum ATX levels. ATX and LPA receptors merit consideration as therapeutic targets of HCC.

## Introduction

Lysophosphatidic acid (1- or 2-acyl-lysophosphatidic acid; LPA) is known as a circulatory lipid mediator and elicits a wide variety of biological responses, including cell migration, angiogenesis, and smooth muscle contraction [1, 2]. Autotaxin (ATX) was originally discovered in conditioned medium from human melanoma cell cultures as a stimulator of cell migration [3]; however, it was later revealed that ATX possesses lysophospholipase D activity [4, 5] to generate LPA in the blood from lysophospholipids [6]. Indeed, LPA levels in plasma from heterozygous ATX-null mice were approximately half those in plasma from wild-type mice [7, 8]. Furthermore, homozygous ATX-null was embryonically lethal in mice [7], suggesting the important role of the ATX-LPA axis *in vivo*.

We have previously explored the potential roles of the ATX-LPA axis in the liver. We first found that LPA stimulates the proliferation of hepatic stellate cells [9], a key player in liver fibrosis. Then, LPA enhances the contractility [10, 11] and inhibits the apoptosis of those cells [12], which suggests that the ATX-LPA axis may be involved in the pathogenesis of liver fibrosis. Then, through *in vivo* experiments that sought to clarify the potential roles of ATX and LPA in liver fibrosis, we found that serum ATX and plasma LPA levels increase with the stage of liver fibrosis in patients with chronic hepatitis C [13, 14] and in those with surgically treated hepatocellular carcinoma (HCC) [15]. A strong correlation between liver fibrosis and serum ATX levels or plasma LPA levels was confirmed in experimental animals, *i*.*e*., in rats with liver fibrosis induced by carbon tetrachloride [16].

Of interest is the fact that HCC commonly develops in the fibrotic liver as a result of chronic liver injury, and patients with cirrhosis with advanced fibrosis are at the highest risk [17]. Because serum ATX and plasma LPA levels increase with liver fibrosis, the majority of HCC cells *in vivo* are likely exposed to an abundance of ATX and LPA. Theoretically, HCC cells with increased LPA receptors could be responsive to such an abundance of LPA. Thus, we hypothesized that LPA might contribute to HCC progression in patients with increased plasma LPA levels and enhanced LPA receptor expression levels in HCC tissue.

In this context, the *in vitro* evidence that suggests that an important role for LPA in HCC has been accumulating. A critical role of LPA in HCC cell motility through Rho and Rho kinase activation was first proposed [18, 19], and the augmentation of human HCC cell invasion by LPA was reported through LPA receptor 1 and MMP-9 expression [20], which implied the contribution of LPA to HCC metastasis or invasion. In contrast, *in vivo* evidence showing the association of LPA with HCC in humans is scarce. The expression of LPA1, 3, and 6 mRNA among the LPA receptors was detected in human HCC tissue using resected human livers, and LPA6 mRNA levels were significantly increased in HCC compared with normal human liver or adjacent non-tumor liver tissue [21]. Nonetheless, to evaluate the hypothesis derived from *in vitro* evidence that LPA might be involved in the pathophysiology of HCC, *in vivo* analysis in humans is needed.

The measurement of LPA levels in the blood is known to be difficult from a clinical laboratory perspective. First, LPA levels in the blood should be measured in plasma to evaluate the clinical significance because LPA is released from platelets [22]. Second and more importantly, LPA levels in plasma samples can become markedly high after sample preparation unless the temperature is strictly controlled, possibly because the coexistence of synthetic ATX and its substrate lysophosphatidyl choline in plasma samples can lead to abundant LPA production [23]. However, ATX levels can be measured in serum and are stable without requiring strict temperature control [23]. Because a strong correlation between serum ATX levels and plasma LPA levels has been observed in humans [13] and rats [16] with liver injury, we used serum ATX levels as a surrogate for plasma LPA levels [14] in the current study.

## Patients and Methods

### Patients

Among HCC patients who were treated in the Hepatobiliary Pancreatic Surgery Division, Department of Surgery, the University of Tokyo Hospital, between January 2013 and October 2014 and provided consent to be enrolled in this study, sufficient quantities of HCC and its adjacent non-tumorous tissue for the analysis of the expression of LPA receptor mRNA were obtained from 58 patients. All enrolled patients underwent liver resection; among these, 36 patients developed primary HCC, and 22 patients exhibited recurrence.

This study was performed in accordance with the ethical guidelines of the Declaration of Helsinki and was approved by the Research Ethics Committee of the University of Tokyo (No.1143). Written informed consent was obtained for the use of samples.

### Measurement of LPA receptors and phosphatidic acid-selective phospholipase A1ɑ (PA-PLA1ɑ) mRNAs

Tumorous and paired non-tumorous tissues were immersed into RNA later solution (Applied Biosystems, CA, USA) just after collection to stabilize and protect RNA according to the manufacturer’s instruction. Briefly, tissues were cut into 0.5 cm in length, kept at 4°C overnight to allow thorough penetration of the tissue, and then transferred to -80°C until starting RNA isolation.

Total RNA of tumorous and paired non-tumorous tissue was extracted using TRIzol reagent (Invitrogen, CA, USA). One microgram of purified total RNA was transcribed using a SuperScript^™^ First-Strand Synthesis System for RT-PCR (Roche Molecular Diagnostics, CA, USA). Quantitative real-time PCR was performed using a SYBR Green PCR Master Mix (Applied Biosystems by Life Technologies, CA, USA). The primer pairs used were as follows: human LPA1, 5’ -GGCTATGTTCGCCAGAGGACTAT-3’ and 5’-TCCAGGAGTCCAGCAGATGATAA-3’; human LPA2, 5’-CGCTCAGCCTGGTCAAGACT-3’ and 5’-TTGCAGGACTCACAGCCTAAAC-3’; human LPA3, 5’-TCCAACCTCATGGCCTTCTT-3’ and 5’-GACCCACTCGTATGCGGAGA-3’; human LPA4, 5’-GTTTCCGCATGAAAATGAGAA-3’ and 5’ -TGGAAAACAAAGAGGCTGAAA-3’; human LPA5, 5’- CTAACCTCGTCATCTTCCTGCT-3’ and 5’-GAAGGAAGACAGAGAGTGGGAGT-3’; human LPA6, 5’-GGTAAGCGTTAACAGCTCCCACT-3’ and 5’- TTTGAGGACGCAGATGAAAATGT-3’; and internal control ribosomal 18s, 5’-GTAACCCGTTGAACCCCATT-3’ and 5’-CCATCCAATCGGTAGTAGCG-3’. Human phosphatidic acid-selective phospholipase A1ɑ (PA-PLA1ɑ) primers and probes (TaqMan Gene Expression Assays) were obtained from Applied Biosystems (Hs00975890_m1). The samples were incubated for 10 min at 95°C, followed by 40 cycles at 95°C for 15 sec and 60°C for 1 min. The mRNA expression level of the target gene was relatively quantified to ribosomal 18s using the 2^-ΔΔCt^ method (Applied Biosystems, User Bulletin No. 2).

### Measurement of ATX

Serum ATX antigen levels were determined in all enrolled patients at 1 day to 1 week prior to surgery using a specific two-site enzyme immunoassay, as previously described [14].

### Patient follow-up and analysis of HCC recurrence

Monthly follow-up was conducted by the assessment of tumor markers (AFP, AFP-L3, and PIVKA-II) and ultrasound. A dynamic CT scan was conducted at 3 and 6 months post-surgery. We defined recurrence as the appearance of new lesions with radiological features typical of HCC as confirmed by at least two imaging methods [24].

### Statistics

All tests of significance were two-tailed, and *P*<0.05 was considered significant. The cumulative incidence of intra- and extra-hepatic recurrence was calculated by the Kaplan-Meier method, and differences among groups were assessed using the log-rank test. A paired t-test was used to analyze differences in mRNAs in tumors and corresponding non-tumorous tissue. Data processing and analysis were performed using SPSS software version 17.0 or 19.0 (SPSS Inc., Chicago, IL, USA).

## Results

### Patient characteristics

Fifty-eight HCC patients who underwent a surgical treatment were analyzed, among whom, 36 patients had primary HCC, and 22 patients exhibited recurrence, as shown in Table 1. There were 46 male and 12 female patients; 11 patients (19.0%) presented with hepatitis B virus infection, and 22 patients (37.9%) presented with hepatitis C virus infection. The median serum ATX level in the patients was 1.068 mg/L, while the median serum ATX level in 120 healthy subjects was 0.700 mg/L [25]; the difference between the two was significant (*P* <0.001, Wilcoxon rank sum test). These previously analyzed, healthy subjects consisted of 46 females and 74 males. Although the median age of these subjects was 41, younger than that of the current patients, there was reportedly the weakly inverse correlation between serum ATX levels and age in male but not in female [25], suggesting higher serum ATX levels in the current patients.

**Table 1:** Patient characteristics.

Parameter	n = 58
Female/Male	12/46
Age (years)	69.4 (64.1–75.3)
BMI (kg/m^2^)	23.0 (20.3–25.9)
Type of hepatitis	
Hepatitis B (%)	11 (19.0)
Hepatitis C (%)	22 (37.9)
Alcoholic (%)	11 (19.0)
Others (%)	14 (24.1)
Primary cases/Recurrent cases	36/22
Tumor size (cm)	2.6 (1.7–6.2)
Number of tumors	
Single (%)	36 (62.1)
More than 2 (%)	22 (37.9)
White blood cell count (×10^3^/μL)	5.25 (4.10–6.20)
Hemoglobin content (g/dL)	13.5 (12.1–14.6)
Platelet count (×10^4^/μL)	15.1 (12.6–18.4)
CRP (mg/dL)	0.07 (0.03–0.16)
Albumin (g/dL)	4.0 (3.7–4.3)
AST (U/L)	32.5 (25.0–51.8)
ALT (U/L)	27.5 (19.0–43.5)
GGT (U/L)	49.5 (30.5–93.8)
Total bilirubin (mg/dL)	0.75 (0.60–0.98)
Creatinine (mg/dL)	0.83 (0.70–0.92)
Triglyceride (mg/dL)	99 (78–143)
Total cholesterol (mg/dL)	177 (150–198)
Fasting blood glucose (mg/dL)	101 (92–117)
HbA1c (NGSP) (%)	5.9 (5.6–6.8)
PT-INR	0.95 (0.91–0.99)
ICGR15 (%)	11.5 (8.2–16.8)
AFP (ng/mL)	7.5 (2.8–47.5)
AFP-L3 (%)	2.1 (0.5–15.6)
PIVKA-II (mAu/mL)	32.0 (16.3–389.8)
Serum ATX level (mg/L)	1.068 (0.836–1.368)
Background liver	
Fibrosis stage 0/1/2/3/4	4/10/12/12/19
Activity grade 0/1/2	9/36/22
Tumor differentiation	
Good (%)	9 (15.5)
Good to moderate (%)	15 (25.9)
Moderate (%)	25 (43.1)
Moderate to poor (%)	7 (12.1)
Poor (%)	2 (3.4)
Microvascular invasion (+)/(−)	14/44

Values are presented as N (%) or medians (P25, P75).

The mean tumor size was 2.6 cm, and 36 patients (62.1%) had a single tumor nodule. Moderately differentiated tumors were predominant (43.1%), and microvascular invasion was observed in 14 patients (24.1%).

### LPA receptors in HCC tissue

**Fig 1:**
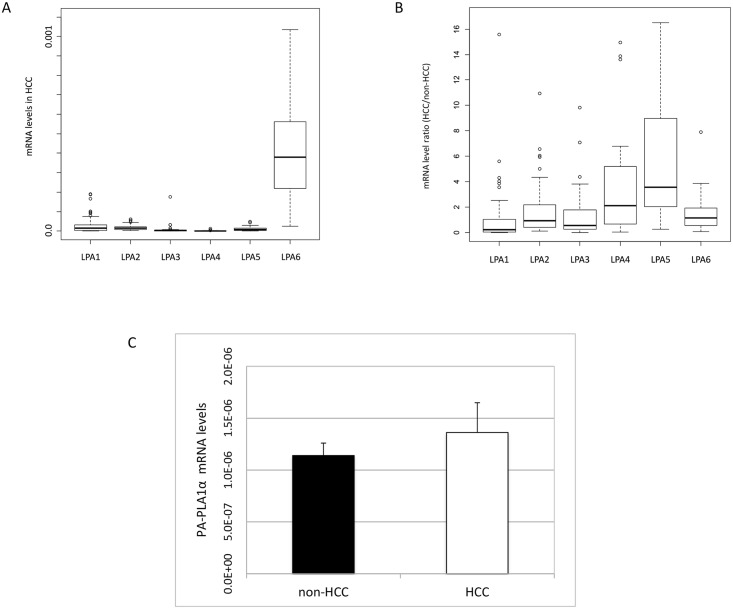
The mRNA expression levels of the LPA receptor and PA-PLA1ɑ, LPA-generating enzyme other than ATX, in HCC. **(a)** mRNA levels of LPA receptors in HCC tissue. **(b)** The ratio of mRNA levels of LPA receptors between HCC and adjacent non-HCC tissue. **(c)** mRNA levels of PA-PLA1ɑ in HCC and adjacent non-HCC tissue.

Fig 1a shows the mRNA levels of the LPA receptors in HCC tissue. LPA6 mRNA levels were the most abundant, followed by LPA1, LPA2 and LPA5 mRNAs, whereas LPA3 and LPA4 mRNAs were virtually absent. Compared with adjacent non-HCC tissue, the ratio of the mRNA levels of HCC and non-HCC tissue was the highest in LPA5, followed by LPA4 and LPA6 (Fig 1b). Thus, in HCC tissue, LPA6 mRNA was expressed most abundantly, and LPA5 mRNA expression was enhanced in HCC compared with non-HCC tissue.

### Relationships between the ATX-LPA receptor axis and HCC profiles

We analyzed the potential association between the ATX-LPA receptor axis and HCC profiles, *i*.*e*., size, number, differentiation or vascular invasion. As shown in Table 2A, 2B and 2C, there was no significant association between tumor size or number and LPA receptors or serum ATX levels. Of note, a significant correlation was observed between LPA2 mRNA levels and HCC differentiation (Spearman’s rho = -0.3152, *P* = 0.018), *i*.*e*., higher LPA2 mRNA levels were associated with poorer HCC differentiation. Furthermore, LPA6 mRNA levels were found to be higher in HCC tissue with microvascular invasion than in those without (*P* = 0.012; Table 3). These results suggest that HCC with higher LPA2 and LPA6 mRNA levels may have a higher malignant potential. In contrast, regarding the ATX-LPA receptor axis and HCC markers in the blood, a significantly positive correlation was found between LPA2 and AFP-L3, LPA6 and AFP, LPA6 and AFP-L3, and ATX and AFP (Table 2A, 2B and 2C).

**Table 2:** Relationships between LPA receptors or LPA-generating enzymes and HCC profiles.

A						
Parameter	LPA1		LPA2		LPA3	
	Spearman’s rho	*P* value	Spearman’s rho	*P* value	Spearman’s rho	*P* value
Tumor size (cm)	-0.0509	0.71	0.0753	0.58	0.0252	0.85
Number of tumors	-0.0241	0.86	-0.0326	0.81	-0.0655	0.63
Degree of tumor differentiation	0.0069	0.96	-0.3152	0.018	-0.1602	0.24
AFP (ng/mL)	0.0888	0.52	0.2117	0.12	-0.0600	0.66
AFP-L3 (%)	0.2622	0.053	0.3458	0.0097	0.0978	0.48
PIVKA-II (mAu/mL)	-0.0558	0.69	-0.1001	0.48	-0.2290	0.10
B						
Parameter	LPA4		LPA5		LPA6	
	Spearman’s rho	*P* value	Spearman’s rho	*P* value	Spearman’s rho	*P* value
Tumor size (cm)	-0.1594	0.25	-0.2174	0.11	-0.0812	0.55
Number of tumors	-0.1019	0.46	-0.0598	0.66	-0.1200	0.38
Degree of tumor differentiation	0.0711	0.61	-0.1737	0.20	-0.1714	0.21
AFP (ng/mL)	-0.0613	0.66	0.0987	0.47	0.3175	0.017
AFP-L3 (%)	-0.0617	0.66	0.2261	0.097	0.2857	0.034
PIVKA-II (mAu/mL)	-0.3252	0.020	-0.1390	0.33	-0.2330	0.096
C						
Parameter	Serum ATX levels		PA-PLA1ɑ			
	Spearman’s rho	*P* value	Spearman’s rho	*P* value		
Tumor size (cm)	-0.1568	0.26	-0.223	0.101		
Number of tumors	-0.0482	0.73	0.097	0.479		
Degree of tumor differentiation	0.3045	0.025	-0.06	0.651		
AFP (ng/mL)	0.3530	0.0088	0.093	0.499		
AFP-L3 (%)	0.2511	0.070	0.222	0.107		
PIVKA-II (mAu/mL)	-0.0544	0.71	-0.246	0.0813		

Spearman’s rank correlation was used to test the associations.

**Table 3:** Relationships between LPA receptors or LPA-generating enzymes and microvascular invasion.

	Microvascular invasion (+)	Microvascular invasion (-)	*P* value
LPA1	1.14×10^−5^	1.46×10^−5^	0.99
	(2.51×10^−6^–3.21×10^−5^)	(4.00×10^−6^–3.10×10^−5^)	
LPA2	2.11×10^−5^	1.34×10^−5^	0.12
	(1.06×10^−5^–3.26×10^−5^)	(6.70×10^−6^–2.00×10^−5^)	
LPA 3	2.39×10^−6^	1.70×10^−6^	0.95
	(3.43×10^−7^–5.87×10^−6^)	(9.00×10^−7^–3.30×10^−6^)	
LPA 4	1.40×10^−6^	7.00×10^−7^	0.14
	(9.00×10^−7^–1.70×10^−6^)	(4.00×10^−7^–1.80×10^−6^)	
LPA 5	7.79×10^−6^	6.70×10^−6^	0.78
	(4.15×10^−6^–1.23×10^−5^)	(3.50×10^−6^–1.51×10^−5^)	
LPA 6	6.10×10^−4^	3.51×10^−4^	0.012
	(3.60×10^−4^–9.10×10^−4^)	(2.05×10^−4^–4.31×10^−4^)	
Serum ATX levels	1.131	1.055	0.97
	(0.801–1.183)	(0.837–1.368)	
PA-PLA1α	5.01×10^−7^	2.88×10^−7^	0.76
	(6.68×10^−8^–1.11×10^−6^)	(2.59×10^−8^–7.40×10^−7^)	

The values are presented as medians (P25, P75). The Wilcoxon rank-sum test was used.

Because LPA is also produced from phosphatidic acid (PA) by PA-PLA1ɑ [26] and LPA generated by PA-PLA1ɑ and LPA6 play pivotal roles in hair follicle development [27–29], we examined PA-PLA1ɑ mRNA expression in HCC. In line with a previous report [30], PA-PLA1α mRNA levels in non-tumorous liver tissue were minimal and did not differ from those in HCC tissue (Fig 1c). There was no association between PA-PLA1ɑ mRNA levels in HCC and HCC differentiation (Table 2C) or microvascular invasion (Table 3).

### Relationships between the ATX-LPA receptor axis and HCC recurrence

**Fig 2:**
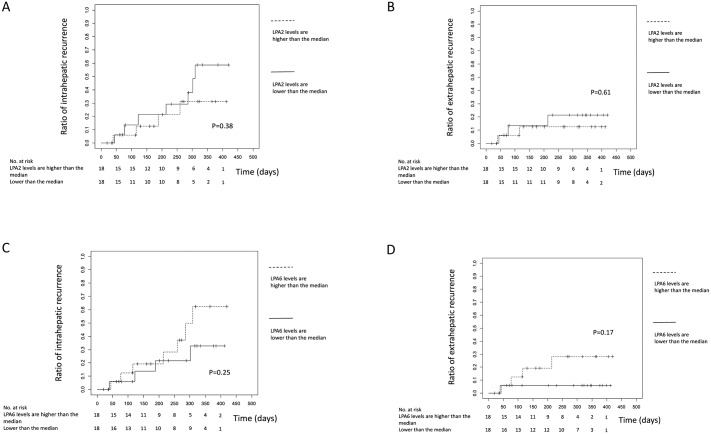
The association of LPA2 and LPA6 mRNA levels with HCC recurrence. The intra- and extra-hepatic recurrence ratio according to the LPA2 mRNA levels **(a and b)** or LPA6 mRNA levels **(c and d)** in HCC.

The observed correlations between the ATX-LPA receptor axis and the pathological profiles of HCC prompted us to examine whether the ATX-LPA receptor axis would be associated with HCC recurrence. Among the enrolled patients, 36 patients with primary HCC were followed up to detect HCC recurrence. During the median follow-up period of 208 days (1st–3rd quartile: 79–316 days), HCC recurred in 11 patients, and HCC recurrence was analyzed according to the levels of LPA receptor mRNA in the original HCC tissue. The cumulative intra- or extra-hepatic recurrence rate estimated by the Kaplan-Meier method did not differ according to mRNA levels of LPA2 or LPA6, as shown in Fig 2a–2d. The mRNA levels of the other LPA receptors (LPA1, 3, 4 and 5) were also not associated with HCC recurrence (data not shown).

**Fig 3:**
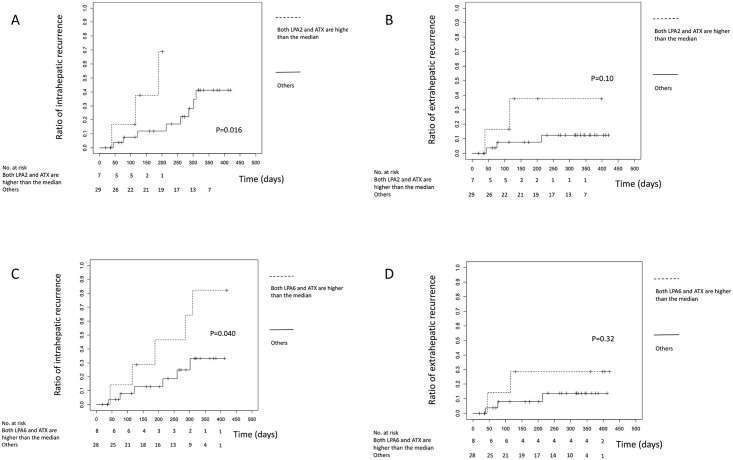
The association of serum ATX levels and LPAR mRNA levels with HCC recurrence. **(a)** Intra- and **(b)** extra-hepatic recurrence ratio of the patients whose LPA2 mRNA levels in HCC and serum ATX levels were higher than the median and other patients. **(c)** Intra- and **(d)** extra-hepatic recurrence ratio of the patients whose LPA6 mRNA levels in HCC and serum ATX levels were higher than the median and other patients.

The magnitude of ligand action is theoretically determined by the quantity of the ligand as well as its receptors. Thus, we next sought to analyze HCC recurrence in consideration of not only LPA receptors but also LPA. As described in the introduction section, serum ATX levels were used as a surrogate for plasma LPA levels [14]. Therefore, we analyzed HCC recurrence by dividing the enrolled patients by serum ATX levels and LPA receptor mRNA levels as follows: patients with serum ATX levels ≥median and LPA receptor mRNA levels ≥median; and other patients. As shown in Fig 3a and 3c, in the analysis of LPA2 or LPA6 and ATX, the cumulative intra-hepatic recurrence rate was higher in the patients with both higher LPA receptors and ATX than the median (*P* = 0.016 and 0.04), and a higher cumulative extra-hepatic recurrence rate was noted in those patients, although the difference was not significant (Fig 3b and 3d). These results suggest that high LPA2 or LPA6 mRNA levels in HCC tissue with high serum ATX levels predict early recurrence.

The false-positive reporting probability (FPRP) values for all findings in Fig 3 were calculated at different prior probability levels. As shown in Table 4, for a prior probability of 0.1, assuming the OR for HCC recurrence was 1.50, the FPRP value was 0.152 for a risk association with patients whose both LPA2 and ATX were higher than the median. All these significant associations tested by FPRP were considered noteworthy, using the criteria of the probability of a false-positive result less than 50%. In contrast, those findings with greater FPRP values may be false positive [31].

**Table 4:** False-positive report probability values for associations of serum ATX levels and LPA2 and LPA6 mRNA levels with HCC recurrence.

Patients group	OR (95% CI)	*P* value	Statistical power	Prior probability				
				0.25	0.1	0.01	0.001	0.0001
Intrahepatic recurrence								
Both LPA2 mRNA levels and serum ATX levels were higher than the median	1.82 (1.08–2.90)	0.016	0.654	0.066	0.152	0.640	0.947	0.994
Both LPA6 mRNA levels and serum ATX levels were higher than the median	1.61 (1.03–2.55)	0.040	0.823	0.169	0.340	0.836	0.980	0.998
Extrahepatic recurrence								
Both LPA2 mRNA levels and serum ATX levels were higher than the median	1.56 (0.88–2.67)	0.10	0.818	0.337	0.562	0.927	0.992	0.999
Both LPA6 mRNA levels and serum ATX levels were higher than the median	1.40 (0.82–2.63)	0.32	0.866	0.575	0.773	0.971	0.997	0.999

The OR and *P* values were reported in Fig 3a, 3b, 3c and 3d. Statistical power was calculated using the number of observations in the study and the OR and *P* values in this table.

In addition, the potential association between PA-PLA1ɑ mRNA levels in HCC and HCC recurrence was analyzed. Firstly, PA-PLA1ɑ mRNA levels in HCC were not associated with HCC recurrence (S1a and S1b Fig). Secondly, when the patients were divided into those with both PA-PLA1ɑand LPA6 mRNA levels ≥median and others, the cumulative recurrence rate was not different between the two groups (S1c and S1d Fig).

## Discussion

In the current study, we sought to examine the potential relevance of ATX and LPA to HCC in humans. A simple analysis regarding LPA receptors levels and pathological profiles of HCC revealed a correlation between higher LPA2 mRNA levels and poorer differentiation and a correlation between higher LPA6 mRNA levels and microvascular invasion, which suggested that HCC with increased LPA2 and LPA6 expression may be associated with a high potential for malignancy. Then, the analysis of recurrence with primary HCC revealed no association between the mRNA levels of LPA receptors and recurrence. However, when serum ATX levels were added to the analysis as a surrogate for plasma LPA levels, we found that higher LPA2 or LPA6 mRNA levels in HCC plus higher serum ATX levels were risk factors for HCC recurrence. These lines of evidence suggest that HCC, likely exposed to abundant LPA due to fibrosis in the background liver, may be responsive to LPA in the context of increased LPA receptor expression, especially LPA2 and 6, to cause recurrence.

As mentioned in the introduction section, the abundance of LPA1, 3 and 6 expression in HCC and the increase in LPA6 expression in HCC compared with non-tumor liver tissue [21] have been previously reported. Furthermore, it has recently been shown that LPA6 overexpression in HCC sustains tumorigenesis and growth and is associated with poor survival [32]. In line with these previous findings, the current study revealed the abundance and up-regulation of LPA6 expression, the correlation of LPA6 expression with microvascular invasion in HCC, and high levels of LPA6 expression in HCC with high serum ATX levels as risk factors for recurrence, which suggested the roles of LPA6 in the malignant potential of HCC. Although the function of LPA6, the most recently characterized LPA receptor subtype, in cancer cells has not yet been fully elucidated, a role of LPA6 in motile and invasive activity has been reported in pancreatic cancer cells [33], in addition to its role in tumorigenesis and growth in HCC cells [32]. LPA6 and LPA generated by PA-PLA1ɑ were shown to play a pivotal role in hair follicle development [27]. Thus, we measured PA-PLA1ɑ mRNA levels in HCC. We found that PA-PLA1ɑ mRNA levels in HCC tissue were not different from those in non-tumorous liver tissue and were not associated with HCC differentiation, microvascular invasion and recurrence, which suggested a minimal role of PA-PLA1ɑ in HCC pathophysiology.

However, we found a significant link between LPA2 and HCC differentiation. To our knowledge, no report in the literature has addressed LPA2 and HCC. Indeed, a previous report on surgically treated HCC revealed minimal levels of LPA2 mRNA in HCC tissue [21]. Thus, the current findings showed that the relatively high LPA2 mRNA levels in HCC correlated with a poorer differentiation of HCC and were a risk factor for recurrence when combined with serum ATX levels. Notably, it has been reported that LPA2 enhances the metastatic potential of ovarian cancer cells [34] and that LPA2 expression is an important process in the carcinogenesis of the stomach [35] and intestine [36]. Thus, LPA2 may be associated with the potential for malignancy in several cancers. Nonetheless, the implications of the high LPA2 expression in HCC are worthy of further evaluation with a larger sample size.

We have recently demonstrated that high serum ATX levels in HCC patients are caused by background liver fibrosis but not by HCC [15]. It has long been known that advanced fibrosis in the background liver of HCC is a risk for poor prognosis in general because poor liver function as a result of advanced fibrosis could aggravate the prognosis of HCC patients [37]. In addition, high serum ATX levels caused by advanced fibrosis could also explain why advanced fibrosis in background liver of HCC is a risk factor for poor prognosis because high serum ATX levels with increased LPA2 or LPA6 expressions in HCC may be associated with early recurrence, as demonstrated in the current study.

We acknowledge the limitations of the current study, mainly that the results are observational with few mechanistic insights. We first aimed to evaluate the hypothesis derived from the accumulated *in vitro* evidence indicating the role of LPA in HCC cell migration or invasion, as *in vivo* evidence of LPA and HCC in humans was relatively scarce. Thus, analyses of LPA and ATX in the blood and LPA receptors in HCC in comparison with non-HCC tissues were conducted in humans. Experiments to provide mechanistic insights were not performed because only human samples from prior operations were used.

In conclusion, LPA2 or LPA6 mRNA levels in HCC and serum ATX levels may be involved in the pathophysiology and recurrence of HCC. The ATX and LPA receptor axis merits consideration as a therapeutic target for HCC.

## Supporting Information

S1 Fig Association of PA-PLA1ɑ mRNA levels with HCC recurrence. **(a)** Intra- and **(b)** extra-hepatic recurrence ratio of the patients according to PA-PLA1ɑ mRNA levels in HCC. **(c)** Intra- and **(d)** extra-hepatic recurrence ratio of the patients whose LPA6 and PA-PLA1ɑ mRNA levels in HCC were higher than the median and those of other patients.
(TIF)
