# Supplementary figures and images for: Higher LPA2 and LPA6 mRNA Levels in Hepatocellular Carcinoma Are Associated with Poorer Differentiation, Microvascular Invasion and Earlier Recurrence with Higher Serum Autotaxin Levels
Source: PLoS One. 2016 Sep 1;11(9):e0161825. doi: 10.1371/journal.pone.0161825 (PMC5008774; doi:10.1371/journal.pone.0161825)

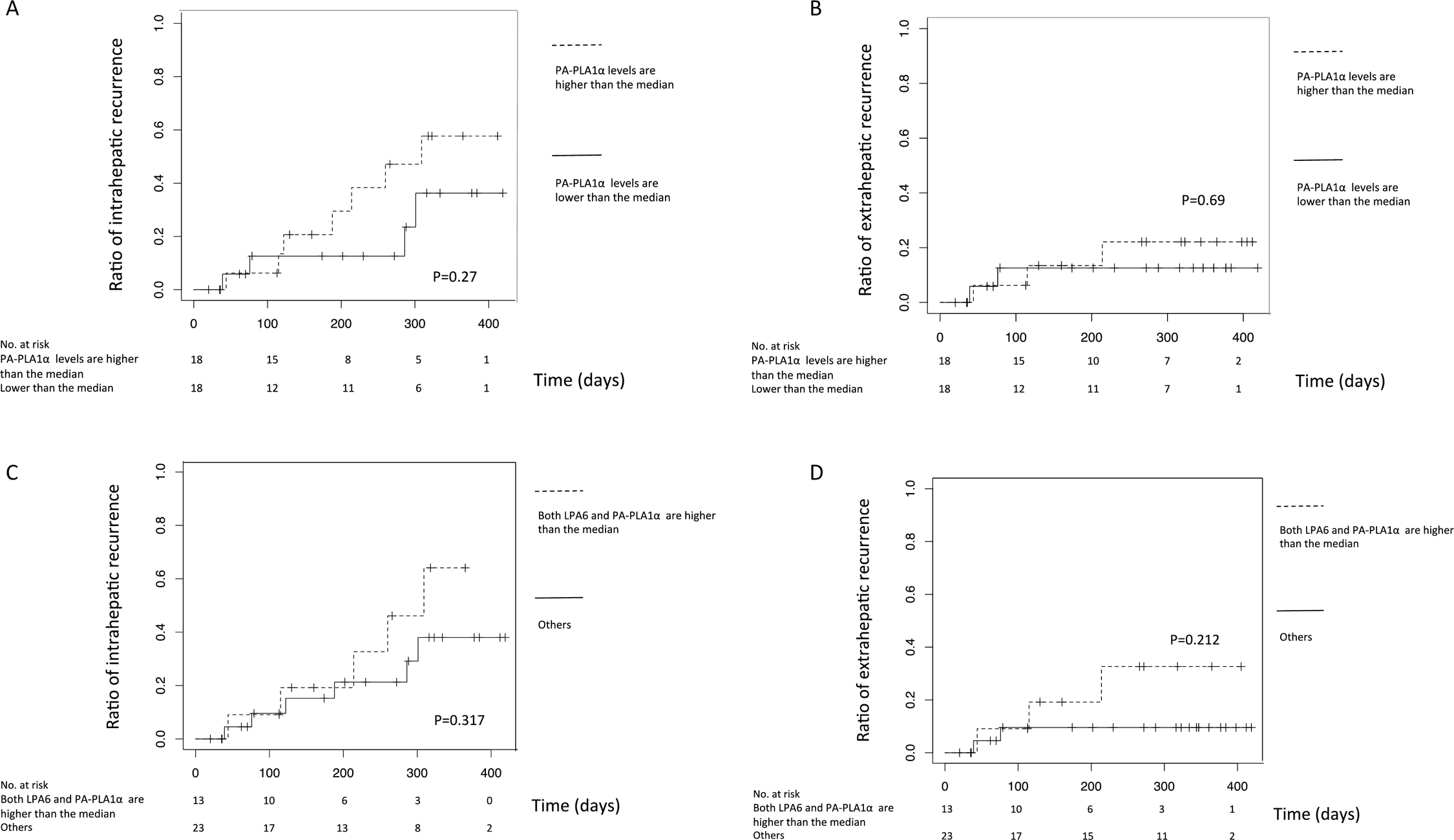

Supplement: S1 Fig — (a) Intra- and (b) extra-hepatic recurrence ratio of the patients according to PA-PLA1ɑ mRNA levels in HCC. (c) Intra- and (d) extra-hepatic recurrence ratio of the patients whose LPA6 and PA-PLA1ɑ mRNA levels in HCC were higher than the median and those of other patients. (TIF) [file pone.0161825.s001.tif]
